# History of Gestational Diabetes Mellitus in Relation to Cardiovascular Disease and Cardiovascular Risk Factors in US Women

**DOI:** 10.3389/fendo.2017.00144

**Published:** 2017-06-26

**Authors:** Derrick C. V. Shostrom, Yangbo Sun, Jacob J. Oleson, Linda G. Snetselaar, Wei Bao

**Affiliations:** ^1^Department of Epidemiology, College of Public Health, University of Iowa, Iowa City, IA, United States; ^2^Department of Biostatistics, College of Public Health, University of Iowa, Iowa City, IA, United States

**Keywords:** gestational diabetes mellitus, cardiovascular disease, cardiovascular risk factors, pregnancy complications, blood lipids

## Abstract

**Background:**

Findings from previous studies examining the association between gestational diabetes mellitus (GDM) and subsequent risk of cardiovascular disease (CVD) have been inconsistent and inconclusive. We aimed to examine the associations of a previous history of GDM with risk of CVD and status of cardiovascular risk factors in a nationwide population-based study in the United States.

**Methods:**

This study included 8,127 parous women aged 20 years or older in the 2007–2014 cycles of the National Health and Nutrition Examination Survey in the United States. The exposure was self-reported diagnostic history of GDM and the outcomes were self-reported diagnostic history of CVD and measurements of cardiovascular risk factors, including blood pressure and blood lipids. Regression models with sample weights were used to examine the associations of GDM with CVD and cardiovascular risk factors.

**Results:**

Among women with a history of both GDM and CVD, CVD was diagnosed on average 22.9 years after the diagnosis of GDM. After adjustment for demographic, socioeconomic, and lifestyle factors, a history of GDM was associated with 63% higher odds of CVD [odds ratio (OR) 1.63, 95% confidence interval (CI) 1.02, 2.62, *p*-value = 0.04]. Further adjustment for body mass index (BMI) modestly attenuated the association (OR 1.52, 95% CI 0.95, 2.44, *p*-value = 0.08). A history of GDM was significantly associated with lower serum level of HDL-cholesterol (adjusted β-coefficient −3.33, 95% CI −5.17, −1.50, *p*-value ≤ 0.001), but not associated with total cholesterol, LDL-cholesterol, triglycerides, or systolic or diastolic blood pressure. Similarly, the association between a history of GDM and HDL cholesterol was attenuated after additional adjustment for BMI (adjusted β-coefficient −1.68, 95% CI −3.38, 0.03, *p*-value = 0.54).

**Conclusion:**

Women with a previous history of GDM have significantly higher risk for developing CVD and lower serum level of HDL cholesterol, compared to women without a history of GDM. The associations may be explained, at least partly, by BMI.

## Introduction

Gestational diabetes mellitus (GDM), a form of glucose intolerance during pregnancy, is a common pregnancy complication affecting approximately 7% (ranging from 1 to 14%) of all pregnancies in the US (1). GDM not only increases short-term risk of adverse pregnancy and birth outcomes but also increases long-term risk of various health outcomes for mothers later in life. A plethora of research has demonstrated that women with a history of GDM are more likely to develop type 2 diabetes. Several studies also suggest that GDM is associated with atherosclerosis (2), metabolic syndrome (3, 4), endothelial and cardiac dysfunction (3), and other intermediate cardiovascular morbidities (5). Based on existing evidence, the American Heart Association has proposed GDM as a major cardiovascular risk factor (6).

Cardiovascular disease (CVD) is the leading cause of death globally (7). Early identification and modification of risk factors has been shown to reduce mortality and morbidity in people with diagnosed or undiagnosed CVD (8). Evidence is still limited on the association between GDM and subsequent risk of overt CVD. Moreover, findings from available studies have been inconsistent and inconclusive (9–12). For instance, Carr et al. found a significantly increased risk of CVD after GDM in women with a family history of type 2 diabetes (9). However, Savitz et al. reported that GDM was significantly associated with the risk of subsequent type 1 and type 2 diabetes, but not CVD outcomes (11). These may be partly due to different settings (population-based vs. hospital-based), population characteristics (e.g., age, race/ethnicity) of participants, and ways of assessment of CVD (self-reports vs. examination) in those studies. Further, some studies examining the association between GDM and CVD appear to be a lack of diversity among participants. These underlie the need for further investigations among the general population with multiple ethnic groups to depict this association (13).

Thus, we sought to determine the risk of developing CVD among women with a history of GDM compared with those without such a history using data from the National Health and Nutrition Examination Survey (NHANES), a nationally representative and diverse population of the United States. Additionally, we compared cardiovascular risk factors between women with and without a history of GDM. We hypothesized that women with a history of GDM would have greater risk of developing overt CVD and unfavorable cardiovascular risk factors compared to women without a history of GDM.

## Materials and Methods

### Study Population

The study population consisted of parous women from the 2007 to 2014 cycles of the NHANES. Briefly, the NHANES, conducted by the Centers for Disease Control and Prevention (CDC), is a large-scale, ongoing, nationally representative health survey of the non-institutionalized US population. NHANES survey data are released every 2 years, with each 2-year cycle consisting of approximately 10,000 participants (14). The surveys comprise population-based, cross-sectional surveys that aim to capture data on diet, nutritional status, general health, disease history, and health behaviors (14). The surveys use multistage, probability clusters to develop a population sample that is nationally representative of the US based on age, sex, and race/ethnicity. In doing so, NHANES proportionally oversamples certain subpopulations of the US in comparison to others to better target the health interests of these subpopulations (15). From 2007 to 2010, NHANES cycles oversampled Hispanic persons, non-Hispanic black persons, low-income non-Hispanic white, and other persons at or below 130% of the federal poverty level (16). The oversampled subpopulations changed slightly in the 2011–2012 and 2013–2014 cycles, with the addition of the low-income non-Hispanic non-Black Asian subgroup, which replaced the non-Hispanic white subgroup in the 2007–2010 cycles (17). NHANES data along with documents on the survey methods and other information are publicly available on the NHANES online website (18). All subjects gave written informed consent in accordance with the Declaration of Helsinki. The study protocol was approved by the NCHS Research Ethics Review Board.

For this analysis, we included female participants, aged 20 years or older, with a prior history of pregnancy. Individuals who reported having a diagnosis of CVD or diabetes present before or during the same year as their diagnosis of GDM were excluded. Finally, we included 8,127 women in this study. The University of Iowa Institutional Review Board has approved this study.

### Exposure Measurement

During the one-time interview through home visit, women were asked “Have you ever been pregnant?” Parous women were further asked, “During your pregnancy, were you ever told by a doctor or other health professional that you had diabetes, sugar diabetes or gestational diabetes?” and “How old were you when you were first told you had diabetes during a pregnancy?” Similarly, the participants were also asked about their diagnostic history and timing for overt diabetes. Based on their response to these questions, parous women were classified as having or not having a history of GDM. Participants who had overt diabetes before the diagnosis of GDM were excluded from the analysis.

### Covariate Assessment

Information on age, race/ethnicity, annual household income, smoking status, and physical activity were obtained during interviews (18). Information on diet was obtained through two 24-h dietary recalls and total energy intake and alcohol intake was calculated using food composition database. Measurements of height, weight, and waist circumference were performed following a standardized protocol, and body mass index (BMI) was computed as weight in kilograms divided by the square of height in meters.

### Outcome Measurement

The primary outcome was CVD, which was self-reported by participants in NHANES during the interview through the following questions: “Has a doctor or other health professional ever told you that you … had 1) congestive heart failure? 2) coronary heart disease? 3) angina/angina pectoris? 4) heart attack? 5) stroke?” and “How old were you when you were first told you had 1) congestive heart failure? 2) coronary heart disease? 3) angina/angina pectoris? 4) heart attack? 5) stroke?” Accordingly, we classified women as developing CVD if they reported having a diagnosis of one or more of these diseases and were classified as not having CVD if they did not report any of these diseases.

Secondary outcomes were CVD risk factors, including blood pressure and blood lipids. Blood pressure, including systolic blood pressure and diastolic blood pressure, was directly measured using standardized protocols. Total cholesterol, triglycerides, and high-density lipoprotein (HDL) cholesterol levels in serum samples were measured using enzymatic methods. Low-density lipoprotein (LDL) cholesterol was calculated from measured values of total cholesterol, triglycerides, and HDL cholesterol *via* the Friedewald’s formula (19): [LDL cholesterol] = [total cholesterol] − [HDL cholesterol] − [triglycerides/5].

### Statistical Analysis

All statistical analyses accounted for the complex, multistage, stratified, and cluster-sampling design (including oversampling of certain subpopulations) of NHANES by using sample weights, strata, and primary sampling units embedded in the NHANES data. Comparisons of baseline characteristics among women with and without a history of GDM were performed using *t* test for continuous variables and the chi-square test for categorical variables.

We used multivariable logistic regression to estimate the odds ratios (ORs) and 95% confidence intervals (CIs) of CVD risk according to history of GDM. We used multivariable linear regression to estimate the β-coefficient and 95% CIs for the associations of history of GDM with blood lipids and blood pressure levels. In multivariable models, we adjusted for age, race/ethnicity, education, family income to poverty ratio, smoking status, alcohol intake, physical activity, total energy intake, and BMI.

Excess adiposity is a strong risk factor for both GDM (20) and CVD (21). Of note, 46.2% of GDM cases was attributable to overweight or obesity (20). Because there was evidence indicating a possible effect modification on the association between GDM and CVD by BMI (22), we performed stratified analyses according to obesity status. In addition, we performed stratified analyses according to hypertension and diabetes status. We conducted interaction tests *via* multiplicative interaction terms in the multivariable models. For the analysis of the secondary outcomes (i.e., blood pressure and blood lipids), we excluded participants who developed CVD and who were currently on medication for hypertension or dyslipidemia. We have checked model assumptions for all the analyses. All analyses were performed using survey procedures of SAS 9.4.

## Results

We identified 787 women who developed CVD among 7,572 women without a history of GDM, and 42 women developed CVD among 555 women with a history of GDM. In the analytical population, women with a history of GDM were more likely to be younger, non-white, more obese, and drink less alcohol (Table 1). Among women with a history of both GDM and CVD, CVD was diagnosed on average 22.9 (SE = 1.8) years after the diagnosis of GDM.

**Table 1:** Characteristics among parous women with or without a history of GDM.

Characteristic	Women without history of GDM	Women with history of GDM	*p*-Value
No. of participants	7,572	555	
Age, years	51.2 (0.25)	44.9 (0.62)	<0.001
Race/ethnicity, %			0.002
Non-Hispanic white	68.5 (1.93)	61.4 (3.35)	
Non-Hispanic black	12.5 (1.04)	11.5 (1.38)	
Hispanic	13.1 (1.26)	18.0 (2.33)	
Other	5.9 (0.46)	9.2 (1.38)	
Education, %			0.43
Less than high school	18.7 (0.88)	19.8 (1.88)	
High school	23.0 (0.75)	20.4 (2.14)	
College or above	58.3 (1.22)	59.8 (2.82)	
Ratio of family income to poverty, %			0.54
≤1.3	22.6 (1.03)	24.7 (1.86)	
>1.3–3.5	33.7 (0.91)	34.7 (2.25)	
>3.5	37.0 (1.29)	37.0 (2.83)	
Missing	6.7 (0.45)	5.4 (1.20)	
Smoking status, %			0.91
Non-smoker	58.1 (0.85)	59.1 (2.63)	
Current smoking	19.2 (0.87)	19.2 (2.15)	
Ever smoker	22.7 (0.90)	21.6 (2.25)	
Alcohol intake, %			0.006
0 g/day	78.3 (0.82)	85.5 (2.08)	
0.1–14.9 g/day	6.1 (0.33)	4.9 (1.20)	
≥ 15 g/day	15.6 (0.70)	9.7 (1.69)	
Physical activity, MET-min/week			0.80
<600	46.1 (0.90)	44.6 (2.91)	
≥600–1,200	12.2 (0.49)	11.8 (1.77)	
≥1,200	41.7 (0.79)	43.5 (2.97)	
Total energy intake (kcal/day)	1,808 (10.6)	1,880 (40.7)	0.09
BMI (kg/m^2^)	29.1 (0.12)	31.7 (0.36)	<0.001
BMI categories, %			<0.001
Normal/underweight	31.3 (0.76)	19.5 (2.02)	
Overweight	30.1 (0.77)	25.8 (2.42)	
Obesity	37.7 (0.82)	54.6 (2.50)	
Missing	0.9 (0.11)	0.1 (0.07)	

*Values are means (SE) or percentages (SE) and are weighted*.

*BMI, body mass index; GDM, gestational diabetes mellitus*.

Compared to women without a history of GDM, women with a history of GDM were more likely to develop CVD, with multivariable-adjusted ORs (95% CIs) of CVD as 1.63 (1.02, 2.62). However, the associations were attenuated and became non-significant after additional adjustment for BMI (Table 2). In a stratified analysis by obesity status, we observed a stronger association between GDM and CVD in obese women [2.16 (1.34, 3.49)], compared to non-obese women [1.58 (1.01, 2.50)] (Table S1 in Supplementary Material). The association did not vary by hypertension status or diabetes status.

**Table 2:** Association between history of GDM and risk of CVD among parous women.

	Women without history of GDM	Women with history of GDM	*p*-Value
Cases of CVD (*n*)	787	42	
Model 1^b^	1.00 (reference)	1.60 (1.02, 2.53)^a^	0.04
Model 2^c^	1.00 (reference)	1.63 (1.02, 2.62)	0.04
Model 3^d^	1.00 (reference)	1.52 (0.95, 2.44)	0.08

*GDM, gestational diabetes mellitus; CVD, cardiovascular disease*.

*Bold values are the values that are statistically significant*.

*^a^Odds ratio (95% confidence intervals)*.

*^b^Multivariable model 1: adjusted for age (years)*.

*^c^Multivariable model 2: multivariable model 1 plus race/ethnicity, education, ratio of family income to poverty, smoking status, alcohol intake, physical activity, and total energy intake*.

*^d^Multivariable model 3: multivariable model 2 plus body mass index*.

In terms of cardiovascular risk factors, women with a history of GDM had lower levels of HDL cholesterol than those without a history of GDM, with adjusted β-coefficient (95% CIs) as −3.33 (−5.17, −1.50) (Table 3). The association did not vary by hypertension status or diabetes status. The association between history of GDM and triglyceride levels differed by hypertension status. Specifically, a history of GDM was not associated with triglyceride levels among women without hypertension; however, among women with hypertension, a history of GDM was associated with higher level of triglycerides, with β-coefficient (95% CIs) as 47.72 (4.78, 90.67) (Table S2 in Supplementary Material). A history of GDM was not significantly associated with total cholesterol, LDL cholesterol, systolic blood pressure, or diastolic blood pressure.

**Table 3:** Association between history of GDM and CVD risk factors among parous women.

		Women without history of GDM	Women with history of GDM	*p*-Value
Systolic blood pressure (mm Hg)	*N*	4,925	398	
	Model 1^b^	Reference	0.20 (−1.24, 1.64)^a^	0.78
	Model 2^c^	Reference	0.16 (−1.34, 1.66)	0.83
	Model 3^d^	Reference	−0.47 (−1.98, 1.04)	0.54
Diastolic blood pressure (mm Hg)	*N*	4,925	398	
	Model 1^b^	Reference	0.77 (−0.56, 2.10)	0.25
	Model 2^c^	Reference	0.93 (−0.41, 2.28)	0.17
	Model 3^d^	Reference	0.53 (−0.81, 1.88)	0.43
Total cholesterol (mg/dL)	*N*	5,599	438	
	Model 1^b^	Reference	−0.19 (−5.08, 4.69)	0.94
	Model 2^c^	Reference	−0.17 (−5.05, 4.70)	0.94
	Model 3^d^	Reference	−0.90 (−5.59, 3.78)	0.70
Triglycerides (mg/dL)	*N*	2,710	199	
	Model 1^b^	Reference	15.37 (−2.23, 32.97)	0.09
	Model 2^c^	Reference	13.44 (−4.06, 30.95)	0.13
	Model 3^d^	Reference	7.11 (−9.86, 24.07)	0.41
HDL-cholesterol (mg/dL)	*N*	5,599	438	
	Model 1^b^	Reference	−4.05 (−6.02, −2.08)	<0.001
	Model 2^c^	Reference	−3.33 (−5.17, −1.50)	<0.001
	Model 3^d^	Reference	−1.68 (−3.38, 0.03)	0.54
LDL-cholesterol (mg/dL)	*N*	2,687	196	
	Model 1^b^	Reference	1.77 (−4.82, 8.37)	0.59
	Model 2^c^	Reference	1.05 (−5.12, 7.22)	0.73
	Model 3^d^	Reference	−0.63 (−6.62, 5.37)	0.83

*GDM, gestational diabetes mellitus; CVD, cardiovascular disease; CI: confidence interval*.

*Bold values are the values that are statistically significant*.

*^a^β-coefficient (95% CIs)*.

*^b^Multivariable model 1: adjusted for age (years)*.

*^c^Multivariable model 2: multivariable model 1 plus race/ethnicity, education, ratio of family income to poverty, smoking status, alcohol intake, physical activity, and total energy intake*.

*^d^Multivariable model 3: multivariable model 2 plus body mass index*.

## Discussion

This study suggests that women with a history of GDM are at greater risk for developing CVD later in life than women without a history of GDM. In addition, women with prior GDM have lower levels of HDL cholesterol and higher levels of triglycerides, compared to women without a history of GDM.

Previous studies on the association of GDM and CVD risk in the general population are sparse. Those studies were conducted in Canada (12, 23, 24), Sweden (22), and France (25). Our results, using data from a nationwide population-based study in the United States were generally consistent with those previous studies. In addition, the magnitude of risk between GDM and CVD presented in this study appears to be similar to that of previous, mostly hospital-based, studies among US women (3, 9, 11, 26). One study in a UK population found a significant association between GDM and calculated CVD risk (based on the Framingham score) in the age-adjusted model, but following further adjustment, the association became non-significant (10).

The associations between GDM and CVD risk factors in this study are consistent with some but not all of previous studies. Zajdenverg et al. found very similar findings with no significant cardiovascular risk factor differences between women with and without a history of GDM but also observed lower levels of HDL cholesterol among women with a history of GDM (27). In some instances, no significant associations between GDM and CVD risk factors have been observed, including no differences in HDL cholesterol (28). Alternatively, other studies have found significant differences in CVD risk factors, with women who have a history of GDM tending to be more obese, have higher blood pressure, and higher triglyceride levels (10, 29, 30).

The underlying mechanisms linking GDM to CVD remain to be elucidated. Obesity is a shared risk factor of GDM and CVD. In this study, we observed a significant association between GDM and CVD among both obese and non-obese women, although the association seemed stronger among obese women compared to non-obese women. Previous studies have shown that altered lipid metabolism, impaired endothelial function, and vascular inflammation may be implicated in the pathogenesis of CVD after GDM (31, 32). Our results indicate that low levels of HDL cholesterol may contribute to the increased risk of CVD in women with prior GDM. Low HDL cholesterol is an established and independent risk factor for coronary artery disease (33) and atherosclerotic CVD (34). In our study, women with a history of GDM reported comparable physical activity levels to women without a history of GDM, but had higher values for BMI and total energy intake. BMI has been inversely associated with HDL cholesterol levels (35). The results in our study also suggested that the association between GDM and low HDL cholesterol levels might be partly explained by BMI. It has been proposed that insulin resistance can cause irregularities in the shapes and sizes of HDL cholesterols, which is an abnormality known to be inversely related to plasma triglyceride levels (35), suggesting that low HDL cholesterol could be secondary to elevated plasma triglyceride levels and increased BMI. It is worth noting that low HDL cholesterol is a more significant risk factor for CVD in women than it is for men (34), which could increase CVD morbidity in women with prior GDM.

The major strength of this population-based study is the use of a nationally representative sample, which facilitate generalization of the findings to the general population in the United States. In addition, with the detailed data collected in the NHANES, we were able to control potential confounding effects from a variety of demographic, socioeconomic, anthropometric, and lifestyle factors. This study has some limitations. First, a history of GDM and CVD diagnosis were both self-reported in NHANES, potentially leading to misclassification of GDM and CVD outcomes. However, previous validation studies have shown high agreement between self-reported and medical record data for both GDM (36) and CVD (37, 38) in US women. Self-report is also applied in NHANES for diet, physical activity, and smoking status. It has been observed that total energy intake was underreported by overweight individuals in previous cycles of NHANES (14), therefore, the USDA’s automated multi-pass method has been employed to reduce misreporting in NHANES 24-h recalls (39). Second, although the sample size was relatively large, we did not have sufficient power to estimate the risk for each of the CVD outcomes. Finally, temporal relationship and reverse causation are common causes for concern in many observational studies, especially in a cross-sectional study setting. However, it is unlikely that reverse causation between GDM and CVD affected our results because the average length of time between a pregnancy complicated with GDM and diagnosis of CVD in our study was 22.9 years. In addition, we excluded cases that reported having a diagnosis of CVD prior to their GDM.

Our findings have important clinical and public health implications. The majority of previous studies involving high-risk populations of CVD focused on older adults. Women who are diagnosed with GDM during pregnancy represent younger, high-risk but usually overlooked population of CVD. For women, the routine screening of GDM during pregnancy offers a unique lifetime opportunity at a young age to reveal their risk of cardiometabolic disorders later in life. Based on the findings of increased risk of CVD after GDM, targeted interventions may be implemented to mitigate the risk at a young age in women with GDM, which could have benefits including but not limited to improved cardiovascular health.

## Conclusion

In a nationwide population-based study in the United States, we show that women with a previous history of GDM have significantly higher risk for developing CVD and lower serum level of HDL cholesterol, compared to women without a history of GDM. These associations may be explained, at least partly, by BMI.

## Ethics Statement

All subjects gave written informed consent in accordance with the Declaration of Helsinki. The study protocol was approved by the NCHS Research Ethics Review Board.

## Author Contributions

DS drafted and revised the manuscript and was responsible for manuscript submission. YS performed data analysis, wrote statistical methods, interpreted results, and critically revised the manuscript. LS and JO interpreted results and critically revised the manuscript. WB conceived the study idea, directed statistical analyses, interpreted results, and critically revised the manuscript. All authors reviewed and approved the final manuscript for submission.

## Conflict of Interest Statement

The authors declare that the research was conducted in the absence of any commercial or financial relationships that could be construed as a potential conflict of interest. The reviewer, ND, declared a shared affiliation, though no other collaboration, with the authors to the handling editor, who ensured that the process nevertheless met the standards of a fair and objective review.
