# Supplementary material for: History of Gestational Diabetes Mellitus in Relation to Cardiovascular Disease and Cardiovascular Risk Factors in US Women
Source: Front Endocrinol (Lausanne). 2017 Jun 26;8:144. doi: 10.3389/fendo.2017.00144 (PMC5483836; doi:10.3389/fendo.2017.00144)
Supplement: Supplementary file 1 [file Table_1.PDF]

Supplementary Table 1. Association between History of GDM and Risk of CVD among Parous Women, According to Obesity Status

		Women without History of GDM	Women with History of GDM	P-value
Cases of CVD		415/4606	13/276	
Non-obese	Model 1[a]	1.00 (reference)	1.50 (0.99, 2.29)[*]	0.06
	Model 2[b]	1.00 (reference)	**1.58 (1.01, 2.50)**	**0.04**
Cases of CVD		384/3239	45/350	
Obese	Model 1[a]	1.00 (reference)	**2.19 (1.45, 3.32)**[*]	**<0.001**
	Model 2[b]	1.00 (reference)	**2.16 (1.34, 3.49)**	**0.002**

Abbreviations: GDM, gestational diabetes; CVD, cardiovascular disease.
Bold values are the values that are statistically significant.
[*] Odds ratio (95% confidence intervals).
[a] Multivariable model 1: adjusted for age (years).
[b] Multivariable model 2: multivariable model 1 plus race/ethnicity, education, ratio of family income to poverty, smoking status, alcohol intake, physical activity and total energy intake.
