# Supplementary material for: History of Gestational Diabetes Mellitus in Relation to Cardiovascular Disease and Cardiovascular Risk Factors in US Women
Source: Front Endocrinol (Lausanne). 2017 Jun 26;8:144. doi: 10.3389/fendo.2017.00144 (PMC5483836; doi:10.3389/fendo.2017.00144)
Supplement: Supplementary file 2 [file Table_2.PDF]

Supplementary Table2. Association between History of GDM and CVD Risk Factors among Parous Women, According to Hypertension Status

		Women without History of GDM	Women with History of GDM	P-value
Triglycerides (mg/dL)				
Without hypertension	N	1754	131	
	Model 1[a]	reference	-2.32 (-15.45, 10.82)[*]	0.73
	Model 2[b]	reference	-4.74 (-17.91, 8.42)	0.47
	Model 3[c]	reference	-9.70 (-22.36, 2.97)	0.13
With hypertension	N	957	69	
	Model 1[a]	reference	**49.05 (5.51, 92.60)**	**0.03**
	Model 2[b]	reference	**47.72 (4.78, 90.67)**	**0.03**
	Model 3[c]	reference	41.82 (-0.87, 84.51)	0.05
HDL-cholesterol (mg/dL)				
Without hypertension	N	3622	297	
	Model 1[a]	reference	**-3.11 (-5.34, -0.88)**	**0.01**
	Model 2[b]	reference	**-2.45 (-4.58, -0.32)**	**0.02**
	Model 3[c]	reference	-1.12 (-3.03, 0.79)	0.25
With hypertension	N	1983	143	
	Model 1[a]	reference	**-5.49 (-8.50, -2.48)**	**<0.001**
	Model 2[b]	reference	**-4.45 (-7.40, -1.51)**	**0.004**
	Model 3[c]	reference	-2.70 (-5.59, 0.19)	0.07

Abbreviations: GDM, gestational diabetes; CVD, cardiovascular disease; CI: confidence interval
Bold values are the values that are statistically significant.
[*] β-coefficient (95% confidence intervals).
[a] Multivariable model 1: adjusted for age (years).
[b] Multivariable model 2: multivariable model 1 plus race/ethnicity, education, ratio of family income to poverty, smoking status, alcohol intake, physical activity and total energy intake.
[c] Multivariable model 3: multivariable model 2 plus BMI.
